# *Prevotella nigrescens* splenic abscess and bacteraemia following laparoscopic sleeve gastrectomy: case report and review of the literature

**DOI:** 10.1186/s12879-026-12936-0

**Published:** 2026-02-27

**Authors:** Mohamed H. Fadul, Zidan Darwish, Soudad Asim Shila, Syed M. Ali, Hamad Abdelhadi

**Affiliations:** 1 Department of Medical Education, Hamad Medical Corporation, Doha, Qatar; 2 Division of Acute Care Surgery, Hamad Medical Corporation, Doha, Qatar; 3 Communicable Diseases Centre, Hamad Medical Corporation, Doha, Qatar; 4 College of Medicine, Qatar University, Doha, Qatar

**Keywords:** Splenic abscess, *Prevotella nigrescens*, Sleeve gastrectomy, LSG, Bariatric surgery, Bacteraemia

## Abstract

**Introduction:**

Obesity is a growing public health priority leading to significant morbidity, mortality, and growing interest in medical and surgical interventions. Due to their perceived long-term efficacy, surgical interventions have gained popularity over the past few decades, supported by recent technical advances. Globally, laparoscopic sleeve gastrectomy (LSG) is the most frequently performed bariatric surgery. Although generally safe, potential post-operative complications include leakage and intra-abdominal infections. Splenic abscesses post-LSG are rare, with only a few cases reported, amongst which *Prevotella nigrescens* was not previously isolated. We describe the rare occurrence of *Prevotella nigrescens* splenic abscess and bacteraemia following LSG.

**Case presentation:**

A 31-year-old female presented three weeks after LSG with fever, rigors, vomiting, and abdominal and shoulder pains. Abdominal ultrasound showed a heterogeneous hypoechoic structure in the superior splenorenal region, and a computed tomography scan reported a 5 cm hypodense septated lesion with subtle peripheral irregular enhancement in the superomedial spleen. A chest x-ray identified a left-sided pleural effusion. Upon CT-guided splenic abscess aspiration, pus was obtained and processed for microbiological evaluation. Eventually, both pus and blood and cultures grew *Prevotella nigrescens.* The patient was treated with antimicrobials and percutaneous aspiration, leading to safe outcomes.

**Conclusion:**

Splenic abscesses are a rare complication of LSG. Patients usually present with febrile symptoms and abdominal pains best evaluated radiologically by CT scans, followed by pathogen identification, guiding safe management approaches. Parenteral antibiotics and source control by percutaneous aspiration are a mainstay of management.

**Clinical trial number:**

Not applicable.

## Introduction

The World Health Organisation (WHO) estimates 2.5 billion adults are overweight and 900 million are obese, more than a threefold increase in prevalence over four decades [1, 2]. This pandemic spans from Western countries to the Gulf region, where the estimated prevalence is up to 70% in subpopulations [3]. Rising interest in obesity interventions stems from various medical complications and reduced life expectancy [2]. Because medical interventions often require more time to demonstrate efficacy, more attention has shifted toward bariatric surgery for more drastic measures.

Amongst surgical bariatric interventions, laparoscopic sleeve gastrectomy (LSG) is one of the leading interventions, being the most frequently performed bariatric surgery worldwide [4]. Although the procedure is generally safe, potential complications include intra-abdominal leakage and infections, postoperative fistulas, and gastric ulcers [5]. Splenic abscesses following LSG are rare, with only a few cases reported; to the best of our knowledge, *Prevotella nigrescens* was not previously isolated in this clinical context. In this case report and literature review, we describe a rare case of a patient who presented with fever and abdominal pain three weeks following an LSG, eventually diagnosed with *Prevotella nigrescens* splenic abscess and bacteraemia confirmed bacteriologically. Following radiological and medical interventions, the case was successfully managed to a safe outcome.

## Case presentation

A 31-year-old female with a past medical history of medically managed scleroderma, body weight of 100 Kg, and a body mass index (BMI) of 37.2 who underwent laparoscopic sleeve gastrectomy (LSG) abroad presented acutely to our hospital. According to the patient, seven days following the surgery, she developed a short febrile illness with fever and palpitations that was managed with a short course of parenteral antibiotics, with resolution of her symptoms, allowing her to travel back. Three weeks following the surgery, she presented to the emergency department (ED) with a two-day history of fever, generalised weakness, dizziness, anorexia, vomiting, and lower abdominal pains. On examination, she was afebrile but tachycardic at 116 beats per minute (bpm) and hypotensive with a blood pressure of 94/62 mmHg. Abdominal examination was unremarkable and, hence, urgent abdominal imaging was not pursued. Laboratory results were unremarkable apart from an elevated C-reactive protein (CRP) level of 63 mg/L. Obtained blood cultures were found to be negative. The patient was treated with symptomatic and supportive measures and received parenteral followed by oral antibiotics (amoxicillin/clavulanate), then discharged with plans for outpatient review.

**Fig. 1:**
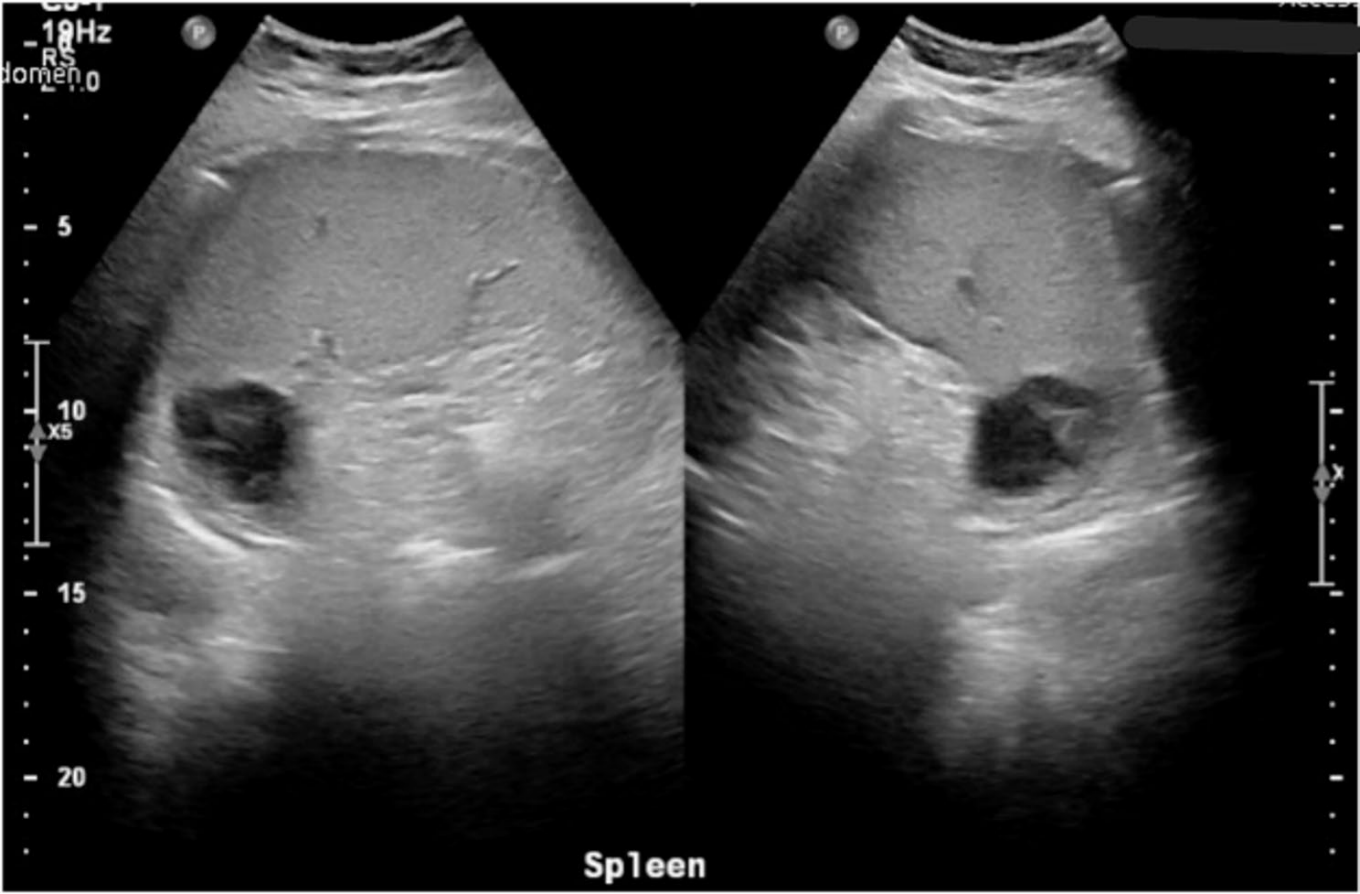
Abdominal ultrasound exam showing a 4 × 3 cm heterogenous hypoechoic structure in the superior splenorenal region

However, a few days later, the patient re-presented with high fever, rigors, abdominal pain, vomiting, anorexia, abdominal pain, as well as shoulder and back pain. This time she was febrile (39.8 °C), tachycardic (138 bpm), and dehydrated. Her blood pressure was 99/60 mmHg while physical examination revealed lower abdominal tenderness and cold extremities. The CRP level rose to 146 mg/L. Abdominal ultrasound showed an enlarged spleen at 14.6 cm with a heterogenous hypoechoic structure seen in the superior splenorenal region measuring about 4 × 3 cm with no associated free fluid in the abdomen (Fig. 1). A chest x-ray revealed a small left-sided pleural effusion.

**Fig. 2:**
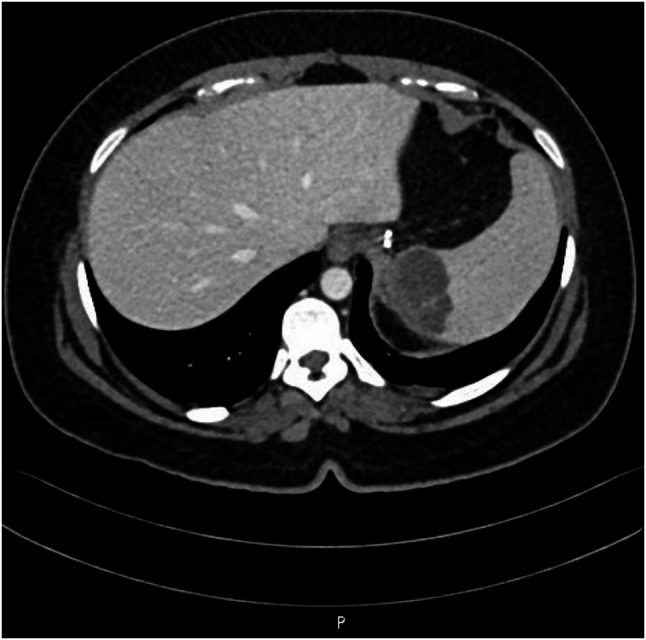
Abdominal CT scan showing a 5 × 2.5 cm hypodense lesion with septation and subtle peripheral irregular enhancement in the superomedial part of the spleen

For further evaluation of the splenic changes, a computed tomography (CT) scan of the abdomen and pelvis with contrast was performed. The stomach was intact with surgical staples along the greater curvature of the stomach, while the superomedial part of the spleen showed a hypodense lesion with septation and subtle peripheral irregular enhancement measuring 5 × 2.5 cm (Fig. 2).

Subsequently, blood cultures obtained at the second presentation grew *Prevotella nigrescens*. Correlating the patient’s history, septic presentation, and high inflammatory markers with the positive radiological and microbiological results, a working diagnosis of a complicated post-LSG splenic abscess was made.

For safe management, the patient was started on parenteral piperacillin/tazobactam 4.5 g four times a day and supportive measures of management, including fluid resuscitation and analgesia. A subsequent barium swallow study demonstrated a satisfactory passage of contrast with no obstruction or leakage. On the same day of admission, the interventional radiology team performed CT-guided aspiration of the splenic abscess, obtaining 13 milliliters of dark brown pus processed for microbiological evaluation with no significant immediate complications. A post-procedure scan showed a small residual collection, most likely due to the loculated nature of the abscess. Cytological and histological evaluation of the splenic collection aspirate showed inflammatory cells and tissues consistent with abscess formation, while the obtained cultures demonstrated a profuse growth of *Prevotella nigrescens* which was sensitive to amoxicillin/clavulanate and metronidazole, identical to blood cultures results.

After completing seven days of piperacillin/tazobactam, the antibiotic was de-escalated to parenteral amoxicillin/clavulanate 1.2 gm three times a day. After clinical improvement and negative blood cultures, she was discharged on oral amoxicillin/clavulanate 875 mg two times a day for five days to complete a total of ten days of amoxicillin/clavulanate. She was later reviewed in the outpatient clinic, where she reported a complete recovery apart from mild residual shoulder pain.

## Discussion

Globally, obesity has reached a pandemic state and has been associated with multiple medical consequences and physical, social, and psychological challenges. Bariatric surgeries, especially laparoscopic sleeve gastrectomy (LSG), have become a popular option for weight reduction and control in recent years because of the long-term results and noticeable rapid weight reduction. The surgical procedure of LSG entails resecting the gastric greater curvature and fundus, where the partial gastrectomy is oriented vertically and parallel to the lesser curvature of the stomach. Although LSG was initially considered a purely restrictive procedure, we now know that it also promotes weight loss by inducing anorexia through the removal of most ghrelin-producing cells located in the gastric fundus [6].

Overall, LSG results in excellent weight loss and remission of most obesity-related comorbidities. Furthermore, because of its technical simplicity and limited alteration of the normal anatomy, it is less drastic with lower morbidity when compared to other bariatric operations such as Roux-en-Y gastric bypass [7]. Currently, the 30-day morbidity and mortality of LSG range from 0 to 17.5% and 0–1.2%, respectively [4]. Nevertheless, LSG complications include intraluminal and intra-abdominal haemorrhage (1–6%), leakage (2–3%), gastroesophageal reflux, and subsequent nutritional deficiencies [5]. A rare but serious complication is the development of visceral abscesses, such as splenic abscess.

Epidemiologically, splenic abscesses are rare, occurring with a prevalence of 0.05–0.7% at autopsies [8]. A systematic review of all reported splenic abscess cases between 1900 and 2022 included 1111 cases managed by combinations of medical, radiological, and surgical interventions [9]. Another systematic review of splenic complications of bariatric surgeries identified 41 articles. Splenic abscess was the most common complication, identified in 44% (*n* = 18) of the cases [10]. A 2022 systematic review of splenic abscesses complicating bariatric surgeries included 27 cases, of which 85% (*n* = 23) were post-LSG splenic abscesses [11]. Specifically for post-LSG splenic abscesses, a 2021 systematic review identified only 18 cases [12].

Aetiologically, splenic abscesses may occur due to diverse pathological mechanisms. The most common is the haematogenous spread from other primary infective foci, such as secondary bacteraemia, infective endocarditis, typhoid and paratyphoid fever, malaria, brucellosis, urinary tract infections, pneumonia, osteomyelitis, and pelvic infections [8]. Another mechanism is the contiguous spread from diverticulitis or pancreatic, retroperitoneal, or subphrenic abscesses. Splenic trauma is another well-recognised aetiologic factor. Splenic infarctions resulting from systemic disorders, such as sickle cell disease, leukaemia, polycythaemia, or vasculitis, can get infected and evolve into splenic abscesses [13]. Alcoholics, diabetics, and immunocompromised patients are among the most susceptible [14]. Iatrogenically, it may occur after splenic artery embolisation procedures [15]. It was also reported as a sequela of COVID-19 infection [16]. As mentioned, it may also complicate gastric surgeries, including LSG. Possible factors include iatrogenic splenic injury during surgery, splenic ischaemia or thrombosis, extension from a gastric staple-line leak, and temporary immune suppression in the immediate postoperative course [12].

Pathogens associated with splenic abscesses are diverse and might vary according to the cause and source of the infection. Streptococci are the most frequent organisms isolated from splenic abscesses [12]. Regarding post-LSG abscesses, isolated organisms include *Staphylococcus aureus*,* Escherichia coli*,* Klebsiella pneumoniae*,* and Streptococcus anginosus*. Furthermore, polymicrobial growth is also reported [12]. In our case, *Prevotella nigrescens*, sensitive to metronidazole and amoxicillin/sulbactam, was isolated from the abscess and the bloodstream. *Prevotella species*, including *P. nigrescens*, are Gram-negative anaerobes that primarily reside in the oral and dental mucosa as well as the gastrointestinal tract from early years of life and are frequently associated with dental infections including tooth decay. Reported secondary infections include bloodstream infections, septic arthritis, and abscesses [17]. To our knowledge, there are no reported cases of *Prevotella nigrescens* splenic abscess.

Fever (94%) and left upper quadrant abdominal pain (56%) were reported to be the most common presenting symptoms in patients with post-LSG splenic abscesses [10]. Although abdominal pain was reported by our patient, its location was somewhat atypical as she initially complained of lower abdominal pain. While our patient presented only 20 days after her LSG, the mean duration from surgery to presentation was reported to be about 98 days [12]. Prompt recognition is vital in limiting morbidity and mortality. White cell counts may show leukocytosis with a left shift, but less commonly in immunocompromised patients. CRP levels are useful in prognosis and monitoring. Positive blood cultures support the diagnosis and predict bacteraemia or septicaemia.

CT scan of the abdomen is a gold standard diagnostic tool with a reported sensitivity of up to 100% [8, 18]. It characteristically shows a low-density lesion that fails to enhance after IV contrast administration. It delineates the size, topography, and access routes to the spleen and surrounding structures. CT-guided drainage can be performed during the examination. Abdominal ultrasonography is often performed before the CT scan and has a lower sensitivity [18]. A follow-up CT scan to re-assess the abscess after conservative or percutaneous treatment might be needed. CXR may reveal a left-sided pleural effusion as in our case [18]. Diagnostic US- or CT-guided percutaneous aspiration may confirm the diagnosis and provide specimens for examination, culture, and sensitivity.

Treatment depends on the patient’s overall condition as well as on the size and topography of the abscess. Treatment options range from conservative options (antibiotics) for small non-loculated abscesses to percutaneous or laparoscopic drainage or even partial or total splenectomy for the less common more complex cases [19]. Empiric broad-spectrum antibiotic therapy, particularly piperacillin/tazobactam, has a primary role in the initial management of splenic abscesses before culture and sensitivity results [12]. The duration of antibiotic use should be judged according to the clinical, biochemical, and radiological response. The presence of bacteraemia or sepsis may require more special considerations. Negative follow-up blood cultures may be required before stopping parenteral antibiotics. Our patient received oral amoxicillin/clavulanate for three days after her first visit to the ED. When admitted on the next visit, she was started on IV piperacillin/tazobactam that was changed seven days later to IV amoxicillin/clavulanate according to sensitivity results.

Percutaneous drainage has gained acceptance as an effective, less invasive treatment modality with high reported success rates [19, 20]. It can also be used as a bridge to elective surgery in temporarily unfit patients. Septated multilocular abscesses may not resolve with percutaneous drainage. Surgical options are reserved for fit patients who are not amenable to or fail percutaneous drainage. Depending on available expertise, laparoscopic or open procedures can be considered. Although not statistically significant, a comparative systematic review and meta-analysis reported lower complication and mortality rates with percutaneous drainage versus splenectomy (10% versus 26%) and (8% versus 12%), respectively [19]. Moreover, percutaneous drainage preserves the spleen and avoids the risks of post-splenectomy complications and the need for long-term prophylaxis.

## Conclusion

Splenic abscesses are generally rare but may complicate intra-abdominal surgical procedures such as LSG. Patients usually present with febrile symptoms and abdominal pain, best evaluated radiologically with CT scans. Upon suspicion, the recommended evaluation is through microbiological identification by blood cultures or radiological interventional aspiration to confirm the diagnosis. Treatment can be conservative with antimicrobials alone or combined interventions with radiologically guided aspiration and rarely drastic interventions through surgical resection or complete splenectomy in complicated cases. Prompt recognition and initiation of appropriate therapy are key steps to safe outcomes.

## Data Availability

All data supporting the findings of this study are available within the paper.

## References

[1] Obesity and Overweight. World Health Organization. https://www.who.int/news-room/fact-sheets/detail/obesity-and-overweight. [accessed 29 November 2024].

[2] GBD 2015 Obesity Collaborators. Health effects of overweight and obesity in 195 countries over 25 years. N Engl J Med. 2017;377(1):13–27. 28604169 10.1056/NEJMoa1614362 PMC5477817

[3] Balhareth A, Meertens R, Kremers S, Sleddens E. Overweight and obesity among adults in the Gulf States: A systematic literature review of correlates of weight, weight-related behaviours, and interventions. Obes reviews: official J Int Association Study Obes. 2019;20(5):763–93. 10.1111/obr.12826. 10.1111/obr.12826 PMC6850146 30653803

[4] Ali M, El Chaar M, Ghiassi S, Rogers AM. American Society for Metabolic and Bariatric Surgery Clinical Issues Committee. American Society for Metabolic and Bariatric Surgery updated position statement on sleeve gastrectomy as a bariatric procedure. Surg Obes Relat Dis. 2017;13(10):1652–7. 29054173 10.1016/j.soard.2017.08.007

[5] Shikora SA, Mahoney CB. Clinical benefit of gastric staple line reinforcement (SLR) in gastrointestinal surgery: a meta-analysis. Obes Surg. 2015;25:1133–41. 25968078 10.1007/s11695-015-1703-x PMC4460272

[6] Abdemur A, Slone J, Berho M, Gianos M, Szomstein S, Rosenthal RJ. Morphology, localization, and patterns of ghrelin-producing cells in stomachs of a morbidly obese population. Surg Laparoscopy Endoscopy Percutaneous Techniques. 2014;24(2):122–6. 10.1097/SLE.0b013e318290167a 24686346

[7] Rosenthal RJ, Panel ISGE. International Sleeve Gastrectomy Expert Panel Consensus Statement: best practice guidelines based on experience of > 12,000 cases. Surg Obes Relat Dis. 2012;8(1):8–19. 22248433 10.1016/j.soard.2011.10.019

[8] Lotfollahzadeh S, Mathew G, Zemaitis MR, Splenic Abscess. [Updated 2023 Jun 3]. In: StatPearls [Internet]. Treasure Island (FL): StatPearls Publishing; 2024 Jan-. Available from: https://www.ncbi.nlm.nih.gov/books/NBK519546/.

[9] Ooi DQH, Ooi JQC, Ooi LLPJ. Splenic abscesses in the new millenium - a systematic review. ANZ J Surg. 2024;94(10):1702–9. 10.1111/ans.19178. 39051445 10.1111/ans.19178

[10] Mousavimaleki A, Amr B, Taherzadeh M, Rokhgireh S, Setaredan SA, Kermansaravi M. Post-Bariatric Splenic Complications; Diagnosis and Treatment. Syst Rev Obes Surg. 2022;32(9):3125–37. 10.1007/s11695-022-06190-x. 10.1007/s11695-022-06190-x 35778627

[11] Buksh MM, Tallowin S, Al Samaraee A. Splenic Abscess Complicating Bariatric Surgery: A Systematic Review. Am Surg. 2022;88(1):28–37. 10.1177/0003134821991971. 33703937 10.1177/0003134821991971

[12] Sakran N, Zakeri R, Madhok B, Graham Y, Parmar C, Mahawar K, Arhi C, Shah K, Pouwels S, Global Bariatric Research Collaborative. Splenic Abscess Following Sleeve Gastrectomy: A Systematic Review of Clinical Presentation and Management Methods. Obes Surg. 2021;31(6):2753–61. 10.1007/s11695-021-05396-9. 33791929 10.1007/s11695-021-05396-9

[13] Al-Salem AH. Splenic complications of sickle cell anemia and the role of splenectomy. Int Sch Res Notices. 2011;2011(1):864257. 10.5402/2011/864257 PMC3200071 22084706

[14] Çulhaci N, Meteoğlu I, Kacar F, Özbaş S. Abscess of the spleen. Pathol Oncol Res. 2004;10:234–6. 15619646 10.1007/BF03033767

[15] Bundy, J. J., Hage, A. N., Srinivasa, R. N., Gemmete, J. J., Srinivasa, R. N., Jairath,N., … Chick, J. F. B. (2019). Intra-arterial ampicillin and gentamicin and the incidence of splenic abscesses following splenic artery embolization: A 20-year case control study. Clinical Imaging. 54, 6–11. 10.1016/j.clinimag.2018.10.005 30476679

[16] AlZarooni N, AlBaroudi A, AlOzaibi L, AlZoabi O. Splenic abscess as a possible sequela of COVID-19: a case series. Ann Saudi Med. 2021;41(5):307–11. 34618603 10.5144/0256-4947.2021.307 PMC8497008

[17] Könönen E, Fteita D, Gursoy UK, Gursoy M. Prevotella species as oral residents and infectious agents with potential impact on systemic conditions. J oral Microbiol. 2022;14(1):2079814. 10.1080/20002297.2022.2079814. 36393976 10.1080/20002297.2022.2079814 PMC9662046

[18] Chiang IS, Lin TJ, Chiang IC, Tsai MS. Splenic abscesses: review of 29 cases. Kaohsiung J Med Sci. 2003;19(10):510–4. 14620677 10.1016/S1607-551X(09)70499-1 PMC11917869

[19] Gutama B, Wothe JK, Xiao M, Hackman D, Chu H, Rickard J. Splenectomy versus imaging-guided percutaneous drainage for splenic abscess: a systematic review and meta-analysis. Surg Infect. 2022;23(5):417–29. 10.1089/sur.2022.072 PMC9208856 35612434

[20] Hadas-Halpren I, Hiller N, Dolberg M. Percutaneous drainage of splenic abscesses: an effective and safe procedure. Br J Radiol. 1992;65(779):968–70. 1450832 10.1259/0007-1285-65-779-968

